# Supplementary material for: MetaQTL: a package of new computational methods for the meta-analysis of QTL mapping experiments
Source: BMC Bioinformatics. 2007 Feb 8;8:49. doi: 10.1186/1471-2105-8-49 (PMC1808479; doi:10.1186/1471-2105-8-49)
Supplement: Additional File 7 — MetaQTL Package : jar file and tutorial. This Zip archive contains both the MetaQTL JAR file and the files of the tutorial. [file 1471-2105-8-49-S7.zip › org.inra.metaqtl/doc/org/metaqtl/class-use/Tree.html]

Uses of Class org.metaqtl.Tree


|  |  |  |
| --- | --- | --- |
| **Overview** \| **Package** \| **Class** \| **Use** \| **Tree** \| **Deprecated** \| **Index** \| **Help** | |  |
| PREV   NEXT | **FRAMES**    **NO FRAMES**     **All Classes** |  |


---


## **Uses of Class org.metaqtl.Tree**

| Packages that use Tree | |
| --- | --- |
| **org.metaqtl** |  |
| **org.metaqtl.algo** |  |
| **org.metaqtl.factory** |  |

Uses of Tree in org.metaqtl

| Fields in org.metaqtl declared as Tree | |
| --- | --- |
| `Tree` | `MetaQtlResult.tree`             The qtl tree |
| `Tree` | `QtlPartition.tree`             The Qtl tree. |

| Methods in org.metaqtl that return Tree | |
| --- | --- |
| `Tree` | `MetaQtlResult.getQtlTree()` |

| Methods in org.metaqtl with parameters of type Tree | |
| --- | --- |
| `void` | `MetaQtlResult.setTree(Tree tree)` |
| `void` | `QtlPartition.setTree(Tree tree)` |

Uses of Tree in org.metaqtl.algo

| Methods in org.metaqtl.algo that return Tree | |
| --- | --- |
| `static Tree` | `HClustAlgorithm.CentroidMethod(double[] x, double[] sd)`             This method performs a hierarchical clustering of the given data points using a centroid method based on the mahalanobis distance. |
| `static Tree` | `HClustAlgorithm.run(double[] x, double[] sd)`             This method performs a agglomerative clustering of the given data points where `x` is the vector of observed values and `sd` the vector of their standard deviations. |
| `static Tree` | `HClustAlgorithm.WardMethod(double[] x, double[] sd)`             This method performs a hierarchical clustering of the given data points using the Ward's method. |

Uses of Tree in org.metaqtl.factory

| Methods in org.metaqtl.factory that return Tree | |
| --- | --- |
| `static Tree` | `TreeFactory.read_newick(java.io.Reader reader)` |
| `static Tree` | `TreeFactory.read_newick(java.lang.String treeStr)` |
| `static Tree` | `TreeFactory.read(java.io.Reader reader)` |
| `static Tree` | `TreeFactory.read(java.lang.String str)` |

| Methods in org.metaqtl.factory with parameters of type Tree | |
| --- | --- |
| `static void` | `TreeFactory.write(Tree tree, java.io.OutputStream stream)`             This method writes out the given tree `nodes` as an array of `TreeNode` on the given output stream stream. |
| `static void` | `TreeFactory.write(Tree tree, java.io.PrintWriter writer)`             This method writes out the given tree `nodes` as an array of `TreeNode` using the given writer writer. |

---


|  |  |  |
| --- | --- | --- |
| **Overview** \| **Package** \| **Class** \| **Use** \| **Tree** \| **Deprecated** \| **Index** \| **Help** | |  |
| PREV   NEXT | **FRAMES**    **NO FRAMES**     **All Classes** |  |


---
